# Swallowing Safety and Efficiency after Open Partial Horizontal Laryngectomy: A Videofluoroscopic Study

**DOI:** 10.3390/cancers11040549

**Published:** 2019-04-17

**Authors:** Nicole Pizzorni, Antonio Schindler, Micol Castellari, Marco Fantini, Erika Crosetti, Giovanni Succo

**Affiliations:** 1 Department of Biomedical and Clinical Sciences “L. Sacco”, University of Milan, Via GB Grassi 74, 20154 Milano, Italy; antonio.schindler@unimi.it (A.S.); micol.castellari@live.it (M.C.); 2 Head and Neck Oncology Service, Candiolo Cancer Institute-FPO IRCCS, Strada Provinciale 142 km 95, 10060 Candiolo (TO), Italy; marcofantini8811@hotmail.it (M.F.); erikacro73@yahoo.com (E.C.); giovannisucco@hotmail.com (G.S.); 3 Department of Oncology, University of Turin, 10043 Orbassano (TO), Italy

**Keywords:** open partial horizontal laryngectomy, supracricoid laryngectomy, dysphagia, swallowing, videofluoroscopy

## Abstract

Dysphagia is common after an open partial horizontal laryngectomy (OPHL). The mechanisms causing lower airways’ invasion and pharyngeal residue are unclear. The study aims to examine physio-pathological mechanisms affecting swallowing safety and efficiency after OPHL. Fifteen patients who underwent an OPHL type IIa with arytenoid resection were recruited. Videofluoroscopic examination of swallowing was performed. Ten spatial, temporal, and scalar parameters were analyzed. Swallowing safety and efficiency were assessed through the Dynamic Imaging Grade of Swallowing Toxicity (DIGEST) scale. Swallowing was considered unsafe or inefficient for a DIGEST safety or efficiency grade ≥2, respectively. Videofluoroscopic measurements were compared between safe vs. unsafe swallowers, and efficient vs. inefficient swallowers. Seven patients (46.7%) showed unsafe swallowing and 6 patients (40%) inefficient swallowing. Unsafe swallowers had worse laryngeal closure (*p* = 0.021). Inefficient swallowers presented a longer pharyngeal transit time (*p* = 0.008), a reduced pharyngoesophageal segment opening lateral (*p* = 0.008), and a worse tongue base retraction (*p* = 0.018 with solids and *p* = 0.049 with semisolids). In conclusion, swallowing safety was affected by incomplete laryngeal closure, while swallowing efficiency was affected by increased pharyngeal transit time, reduced upper esophageal sphincter opening, and incomplete tongue base retraction. The identified physio-pathological mechanisms could represent targets for rehabilitative and surgical approaches in patients with dysphagia after OPHL.

## 1. Introduction

Open partial horizontal laryngectomies (OPHLs) are conservative surgical techniques aimed to the treatment of laryngeal carcinomas in early-intermediated T stage [1]. By contrast with total laryngectomies, the main laryngeal functions (i.e., respiration, phonation, and swallowing) are preserved, thanks to the sparing of at least one functioning crico-arytenoid unit with the corresponding arytenoid and the intact inferior laryngeal nerve of the same side; therefore, the need for a permanent tracheostoma is avoided. Among the OPHLs, OPHL type II is characterized by the resection of the entire thyroid cartilage, with the inferior limit represented by the upper edge of the cricoid ring. Different types of OPHL type II exist, differentiated by the amount of supraglottis removed, and their extension to include one arytenoid (+ARY). In OPHL type IIa, the thyrohyoid membrane is entered horizontally from above, and the pre-epiglottic space and epiglottic cartilage are transected so that the suprahyoid part of the epiglottis is spared. On both sides, the inferior constrictor muscles are incised, the piriform sinuses dissected, the inferior horns of thyroid cartilage cut and the ventricular and vocal folds divided down to the lower limit of resection in the subglottic region. The trachea is mobilized by blunt dissection along the anterior tracheal wall, and a cervico-mediastinal release of the trachea is performed. The cricoid is pulled up to the level of the hyoid bone to achieve the laryngeal reconstruction by a cricohyoidoepiglottopexy.

Swallowing is a complex sensorimotor behavior involving the coordinated contraction and inhibition of the musculature of the mouth, the tongue, the pharynx, the larynx, and esophagus bilaterally in a short interval (0.6–1.0 s) [2]. During the oral and the pharyngeal phase of swallowing, different events occur under voluntary or involuntary control. The timing of swallowing events and the intensity of muscular contraction are modulated based on the characteristics of the bolus to swallow, thanks to the sensory-motor integration at the level of the central pattern generator in the brainstem. In case of the failure of the occurrence of a swallowing event, or of an aberrant sequence, timing, and intensity of these events, swallowing safety and efficiency may be impaired. Safety refers to the ability to transfer the bolus from the mouth to the stomach without penetration or aspiration into the lower airways; efficiency refers to the ability to transfer the bolus from the mouth to the stomach without post-swallow pharyngeal residue [3]. Pulmonary complications (e.g., aspiration pneumonia) and nutritional complications are consequences of impaired swallowing safety and efficiency, respectively. Moreover, swallowing complications comprise reduction of quality of life, limitations to social participation, and negative affective responses [4].

Swallowing function after OPHL type II has been extensively investigated in the literature [5,6]. The incidence of dysphagia is approximately 100% immediately after surgery, but, usually, swallowing function recovers spontaneously in 3 to 6 months post-operatively, with the majority of the patients achieving a free oral diet [6]. Nevertheless, chronic aspiration, especially with liquids, and post-swallow residue, especially with solids, are often detected even in the long-term, and increase the risk of aspiration pneumonia and death [7]. Studies investigating swallowing function in patients who underwent an OPHL mainly focus on signs of dysphagia (i.e., penetration, aspiration, residue). However, there is a paucity of studies assessing the mechanisms causing these signs. In 1996, Woisard et al. [8] analyzed the pathophysiology of swallowing in 14 patients one year after OPHL. Several mechanisms were found, the most frequent being reduced tongue base retraction, reduced laryngeal elevation, reduced laryngeal anteriorization and faulty in the backward movement of the epiglottis [8]. However, the mechanisms underlying reduced safety and efficiency were not investigated. In 2005, Yücetürk et al. used videofluoroscopy to assess swallowing in 10 patients who underwent an OPHL type IIb (with the resection of the whole epiglottis) at least 6 months after surgery [9]. Nine spatial and one temporal measures were analyzed and compared to those of 13 healthy controls. Results showed a statistically significant difference between the two groups for the hyoidomandibular distance during swallowing and at rest, higher in patients than in controls, and for the hyoidovertebral distance during swallowing, lower in patients than in controls. Due to the small sample size and the low number (2/10) of patients with aspiration, no comparisons were made between patients with and without signs of dysphagia. In 2008, Lewin and colleagues assessed swallowing outcomes in 27 patients who underwent an OPHL type II using videofluoroscopy [10]. Patients were on average assessed at 4 weeks after the surgery and re-assessed after 7 weeks from the first videofluoroscopic study. Three mechanisms (hyolaryngeal excursion, tongue base retraction, and neoglottic competency) were rated as normal or impaired. No temporal or biomechanical objective measurements were gained. At the first assessment, reduced hyolaryngeal excursion was identified in 45% of the patients, decreased base of tongue retraction in 27% of the patients, and neoglottic incompetence in 100% of the patients. Results were stable at the second assessment. To the best of our knowledge, no studies investigated the association between mechanisms and the presence of signs of dysphagia in patients with an OPHL type IIa. Therefore, mechanisms causing lower airways’ invasion and post-swallow pharyngeal residue in this population are still unclear.

The study aims to examine videofluoroscopic variables associated with the impairment of swallowing safety and efficiency after OPHL type IIa+ARY. Based on the previous studies, the hypothesis is that the hyoidomandibular and the hyoidovertebral distances during swallowing, the tongue base retraction, and the laryngeal closure may be significantly impaired in patients with unsafe or inefficient swallowing compared to those with safe and efficient swallowing. The knowledge of the mechanisms causing dysphagia in the long-term will provide a basis to identify targeted and effective rehabilitative and surgical strategies to improve functional outcomes, potentially reducing the rate of pulmonary complications and the impact of quality of life.

## 2. Results

**Figure 1:**
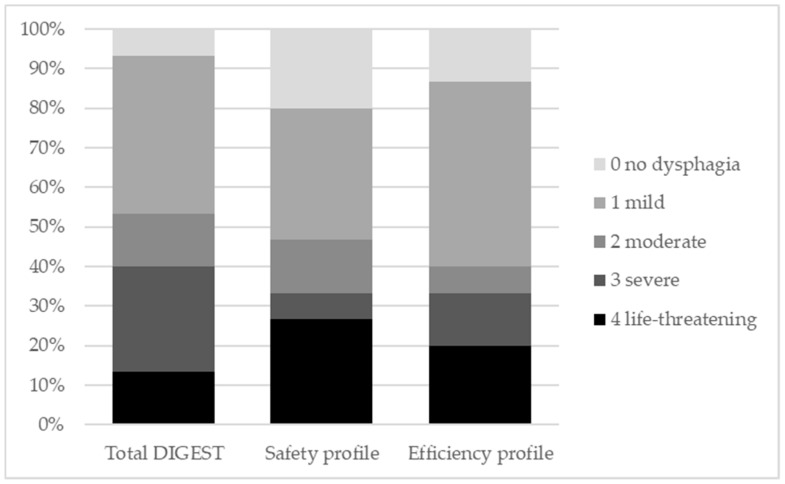
Distribution of the 15 patients with open partial horizontal laryngectomy (OPHL) type IIa and extension to include one arytenoid (+ARY) in the Dynamic Imaging Grade of Swallowing Toxicity (DIGEST) levels.

**Figure 2:**
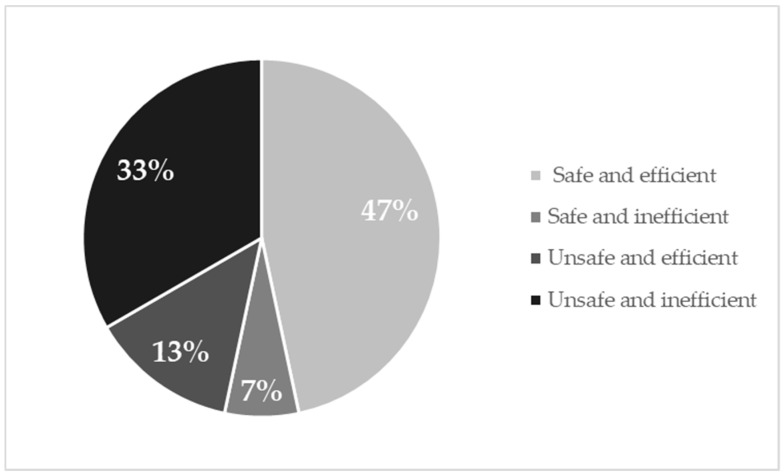
Swallowing patterns of the 15 patients with OPHL type IIa+ARY.

Based on the Dynamic Imaging Grade of Swallowing Toxicity (DIGEST) scores [11], 7 (46.7%) patients who underwent an OPHL type IIa+ARY showed unsafe swallowing (DIGEST safety profile ≥2) and 6 (40%) patients had inefficient swallowing (DIGEST efficiency profile ≥2). Only 1 patient had no signs of dysphagia (total DIGEST score 0) at the videofluoroscopic assessment of swallowing. The distribution of the 15 patients in the DIGEST levels is reported in Figure 1. Four patterns of swallowing proficiency were identified and depicted in Figure 2.

### 2.1. Swallowing Safety: Comparison of Videofluoroscopic Variables

Patients with safe swallowing had comparable age (median 65 IQ range 11.5 vs. median 71 IQ range 15, *p* = 0.463), follow-up period (median 20 months IQ range 22.50 vs. median 8 months IQ range 15, *p* = 0.152), and T stage of the tumor (median 2.5 IQ range 1 vs. median 2 IQ range 2, *p* = 0.694) to patients with unsafe swallowing. Comparisons of videofluoroscopic measures between patients with safe and unsafe swallowing are reported in Table 1. A significant difference was found only for the laryngeal closure (LC) with liquids and solids. Patients with unsafe swallowing showed a more impaired laryngeal closure during swallowing than patients with safe swallowing.

### 2.2. Swallowing Efficiency: Comparison of Videofluoroscopic Variables

Analogously to the safety analysis, patients with efficient swallowing had comparable age (median 66 IQ range 12 vs. median 67.5 IQ range 15, *p* = 1), time from surgery to follow up (median 21 months IQ range 20 vs. median 7 IQ range 9, *p* = 0.066), and T stage of the tumor (median 2 IQ range 1 vs. median 2.5 IQ range 1, *p* = 0.456) to patients with inefficient swallowing. Comparisons of videofluoroscopic parameters between patients with efficient and inefficient swallowing are shown in Table 2. Significant differences were found for 4 videofluoroscopic measures; patients with inefficient swallowing had a longer total pharyngeal transit time (TPT) with semisolids, a narrower pharyngoesophageal segment opening lateral (POL) with semisolids, and an incomplete tongue base retraction (TBR) with semisolids and solids. A trend was recorded for a prolonged pharyngoesophageal segment opening duration (POD) with semisolids, an increased hyoidomandibular distance during swallowing (HMS) with liquids, and a reduced LC with semisolids in patients with inefficient swallowing compared to those with efficient swallowing.

## 3. Discussion

This study firstly investigated mechanisms underlying impairment of the safety and the efficiency of swallowing in patients with OPHL type IIa+ARY, through the analysis of temporal, spatial and ordinal videofluoroscopic measurements. Thus, it provides a better understanding of the physio-pathological changes of swallowing in this population and their clinical relevance.

The age and the time from surgery to videofluoroscopic assessment were compared between patients with impaired safety or efficiency and patients with functional swallowing. The age was comparable in patients with safe vs. unsafe swallowing, and patients with efficient vs. inefficient swallowing. The literature shows inconsistent findings on the effect of age on functional outcomes after OPHL. Two studies have demonstrated no significant influence of age at surgery on swallowing function [12,13]. On the other hand, Benito and colleagues demonstrated that the risk of aspiration increases in patients who underwent an OPHL >70 [14]. Analogously, Naudo and colleagues reported a significant association between age and aspiration [15]. Time from surgery to follow-up did not significantly differ in both the safety and the efficiency comparisons. However, a trend for a longer follow-up period was recorded in the safe and efficient groups. Although patients had at least a 5 months follow-up period, and studies in the literature showed that the recovery of swallowing is completed within 3–6 months after surgery in most if the cases [5,6], it can be speculated that compensatory mechanisms may consolidate even after this time period. Nevertheless, to the best of our knowledge, no longitudinal studies instrumentally assessing the progression of swallowing function after an OPHL from the early post-surgery to the long-term period exists. Only Lewin et al in 2008 performed 2 consecutive videofluoroscopic evaluations after an OPHL type II, but the follow-up period was on average 4 weeks at the first assessment and 7 weeks at the second assessments [10].

Swallowing safety was impaired in the 46.7% of the sample, with a statistically significant difference only for the laryngeal closure parameter. Patients with unsafe swallowing had poorer laryngeal closure. Normally, the closure of the laryngeal vestibule during swallowing is achieved thanks to the concomitant epiglottic inversion, hyo-laryngeal elevation, aryepiglottic fold bunching, arytenoid adduction, base of the tongue posterior movement, and pharyngeal constriction [16,17,18]. Due to the anatomical changes of this district, after an OPHL type IIa+ARY, the sphincteric action of the neolarynx is provided by the approximation of the mobile arytenoid cartilage (rotating forward and inward) and the epiglottis (tilting backward) [8]. Other configurations are described in the literature but are rarely observed [19]. Analogously to our findings, an inadequate closure of the laryngeal vestibule entry was observed by Logemann et al in patients who were not eating at 2 weeks after an OPHL type I (or supraglottic laryngectomy), when compared to the patients who restored oral feeding at the same time-point [20]. Indeed, they identified two critical factors in the recovery of swallowing: (a) the airway closure at the laryngeal entrance (i.e., the space between the arytenoid cartilage and the base of the tongue), and (b) the contact of the base of tongue with the posterior pharyngeal wall.

The closure of laryngeal vestibule may be targeted through both a swallowing therapy and surgical rehabilitative approaches. Supraglottic and super-supraglottic maneuvers are two breath-holding swallowing maneuvers aiming to improve the extent and the duration of the laryngeal vestibule closure. Their efficacy on both swallowing kinematics and the rate of laryngeal penetration and aspiration was proved in a cohort of patients with oropharyngeal dysphagia from different etiologies and a cohort of patients with radiation-induced dysphagia [21,22]. Surgical approaches comprise the endoscopic injection of different materials into the preserved arytenoid or into the superior face of the cricoid ring [23]. The choice of the most appropriate injection site and the material is based on a careful fiberoptic endoscopic examination of swallowing. Preliminary results from a case series of 7 patients with an OPHL type IIa+ARY showed a complete recovery of the lower airways’ protection during swallowing in 4 patients, and a partial recovery with occasional aspiration with liquids in 2 patients [23].

Swallowing was considered inefficient in the 40% of the sample. Patients with inefficient swallowing had a longer total pharyngeal transit time, a narrower upper esophageal sphincter (UES) lateral opening, and a poorer contract between tongue base and posterior pharyngeal wall. An interaction between these mechanisms can be found, highlighting their cooperation in reducing swallowing efficiency. Indeed, the opening of the UES not only relies on the inhibition of the cricopharyngeus muscle’s contraction but also on the generation of adequate pharyngeal pressures and the anterior-superior motion of the hyolaryngeal complex [24,25,26]. Pharyngeal pressures depend on the action of the velopharyngeal valve, the protrusion of the tongue base, and the contraction of pharyngeal constrictors [27]. Pharyngeal pressures influence the pharyngeal transit time [28]. Therefore, it can be speculated that incomplete tongue base retraction resulted in a reduced pharyngeal pressure, prolonging the duration of the total transit time and reducing the UES lateral opening. The reduced UES opening and the incomplete tongue base retraction lead to post-swallow residue in pyriform sinuses and valleculae. As previously stated, hyolaryngeal excursion contributes to UES opening and its impairment was identified as a mechanism of dysphagia in the OPHL population by several studies [8,9,10]. In the present study, a trend toward an increased hyoidomandibular distance during liquid swallows coupled with a similar hyoidomandibular distance at rest was observed in patients with inefficient swallowing compared to patients with efficient swallowing, suggesting a decreased hyoid elevation in the former group. Further studies, including larger number of patients are needed to analyze this parameter in detail.

No studies have assessed the association between videofluoroscopic measurements and post-swallow residue in patients after OPHL; however, studies exist on other populations. Pauloski and colleagues highlighted an association between a reduced tongue base or posterior pharyngeal wall movement and the pharyngeal residue in patients with head and neck cancer after the completion of radiotherapy [29]. Another study on patients with oropharyngeal dysphagia found a reduction of the mean peak pharyngeal pressure in patients with an incomplete tongue retraction, and a strong asssociation with the presence of post-swallow pharyngeal residue [30]. Interestingly, in the present study a statistically significant difference in tongue base retraction was found only for semisolids and solids, but not for liquids. This finding is in accordance with a study by Pouderoux and Kahrilas showing a modulation of swallowing pressure at the level of tongue in response to varying bolus volumes and textures [31]. In particular, an increase of the viscosity was associated with an increased tongue pressure, thus, explaining the association between a deficit in tongue base retraction and pharyngeal residue with solids. In swallowing therapy, the Shaker head lift exercise [32] and the Mendelsohn maneuver [33] are strengthening exercise to increase the UES opening in patients with oropharyngeal dysphagia. Moreover, the effortful swallow [34] and the tongue hold swallow [35] were found to improve the contact between the base of the tongue and the posterior pharyngeal wall. Martin-Harris and colleagues have investigated the effect of a respiratory-swallowing training in patients with head and neck cancer treated with different modalities and chronic dysphagia and have reported an improvement in the laryngeal closure and the tongue base retraction, associated with a reduction of lower airways’ invasion and pharyngeal residue [36]. As for laryngeal closure, fat injections have been proposed in patients who underwent an OPHL type I for the correction of the tissue loss at the level of the base of tongue, with promising results on the improvement of the swallowing efficiency [37,38].

In the present study no preoperative assessment was performed because of the observational design. Different data on swallowing kinematics in healthy subject using videofluoroscopy were reported in the literature [9,25,39]. Concerning the temporal measures, in the present study the median pharyngeal transit time ranged between 0.32 and 0.42 s, whereas the median duration of UES opening was between 0.24 and 0.28 s. These durations are lower than those reported by Molfenter and Steele in a metanalysis on healthy subjects, which were on average 0.84 and 0.46 s, respectively [39]. The UES lateral opening was reported to range between 8 and 9 mm in healthy subjects; this measurement was slightly higher in the present OPHL type IIa sample (median 8–12 mm). Yücetürk and colleagues analyzed the position of the hyoid bone in a sample of 13 volunteers using 10 mL of liquids and reported a mean value of 41 mm for the HMS, 22 mm for the hyoidomandibular distance at rest (HMR), and 42 mm for the hyoidovertebral distance during swallowing (HVS) [9]. In the present study median HMS, HMR, and HVS with liquids were 0–6 mm, 24–33 mm, and 58–64 mm, respectively. However, the high variability introduced by factors related to the participant (e.g., age and height), to the stimulus (e.g., bolus consistency and volume), and to the procedure (e.g., frames/second and cueing) limits the possibility to compare the videofluoroscopic measures of the patients included in the present study with those of healthy controls from other studies.

The DIGEST was used for the first time to assess swallowing function in a sample of patients who underwent and OPHL. Indeed, the scale was specifically developed for patients with head and neck cancer, but was validated only in patients treated with surgical and non-surgical organ preservation modalities [11]. Difficulties in the application of the DIGEST to the OPHL populations may be related to the anatomical changes induced by the resection and reconstruction process, which alters the landmarks normally used to identify the presence of lower airways’ invasion. However, in the OPHL type IIa population included in the present study, the main anatomical landmarks (i.e., the epiglottis and the neoglottis) were maintained or easily recognizable on a lateral plane view.

The strengths of the study are the highly homogeneous cohort and the use of objective measures for the study of swallowing mechanisms. Only patients who underwent an OPHL type IIa+ARY, which is the most performed type of OPHL [40] in our caseload, were included. Objective videofluoroscopic measures are reliable and repeatable, reducing the subjectivity related to the use of perceptual variables. Although the fiberoptic endoscopic evaluation of swallowing and the videofluoroscopy are both considered gold-standard for the assessment of swallowing function [41], only the videofluoroscopy can allow to investigate the pathophysiological mechanisms causing signs of dysphagia.

Nevertheless, the study has some limitations. The sample size is limited to 15 patients. The sample size in comparable or even larger than other studies in the literature assessing swallowing mechanisms after an OPHL [9,10]. However, the statistical power may be inadequate to highlight some of the differences that were found not to be statistically significant in the study but that showed a trend toward it. Moreover, a long-term follow-up was conducted in all patients and the time from surgery to follow-up did not significantly differ between patients with safe and efficient swallow and patient with unsafe and inefficient swallow, but a trend for longer follow-up was found in patients with safe and efficient swallowing. Although the distance between surgery and videofluorosocopic assessment may represent a potential bias to the results of the present study, the extent of this bias cannot be assessed. Indeed, no data are currently available on the long-term progression of the recovery of swallowing function after an OPHL. Therefore, future studies with longer follow-up and longitudinal studies are recommended and the results here presented should be interpreted with caution.

Future studies may expand the analysis of the mechanisms affecting swallowing safety and efficiency to other types of OPHL. High-resolution impedance manometry and simultaneous endoscopic and videofluoroscopic assessments of swallowing may provide a deeper understanding of these mechanisms. Interventional studies should be performed to verify the efficacy of rehabilitative and surgical strategies targeting the identified mechanisms on swallowing safety and efficiency in patients with an OPHL type IIa+ARY. Moreover, the impact of an impaired safety and efficiency of swallowing on the onset of pulmonary complication and the reduction of quality of life should be investigated, together with the role of other relevant aspects, such as the type oral intake, the oral hygiene, and patient-reported outcomes.

## 4. Materials and Methods

The cross-sectional study was carried out according to the Declaration of Helsinki. All subjects enrolled in the study gave their written informed consent; all data were collected prospectively. In this cross-sectional study, patients were routinely treated and assessed according to their pathology and in accordance with the Italian guidelines [42]. Swallowing assessment was performed in patients who reported swallowing complaints and, therefore, assessed within the normal clinical routine. For these reasons, ethical approval is not available.

### 4.1. Patients

Patients were recruited at the Otorhinolaryngology Service of the Martini Hospital (Turin, Italy) during their follow-up assessment, over a 5 months period. Selection criteria were: OPHL type IIa+ARY, subjective swallowing complaints, no evident disease at the last follow-up, preservation of respiration and speech, absence of a tracheostomy, no salvage total laryngectomy performed and time from surgery to follow-up between 3 months and 3 years. For the homogeneity of the sample, only male patients who underwent an OPHL type IIa+ARY were included. Fifteen patients were recruited. Median age was 67 (range 52–82), the median time from surgery to the last follow-up was 17 months (range 5–34). Tumors’ stage was T2N0 tumor in 8 patients, T3N0 in 6 patients, and T4N0 in 1 patient. Only one patient underwent radiotherapy after surgery.

An endoscopic assessment of the movement of the spared arytenoid was performed postoperatively as part of the routine clinical practice to exclude the presence of an ankylosis of the cricoarytenoid joint. The arytenoid movement was preserved in all the patients.

### 4.2. Videofluoroscopic Study of Swallowing

Patients underwent a standardized videofluoroscopic assessment of swallowing with the Digital Subtraction Angiography Unit (Advantix LC Plus, General Electric) at 25 frames/second. Patients were seated in the lateral viewing plane. Videofluoroscopic studies were digitally recorded, downloaded, and de-identified for subsequent data analyses. A liquid 10 mL barium bolus, a semisolid 10 mL barium bolus, and half biscuit were administered.

### 4.3. Dynamic Imaging Grade of Swallowing Toxicity (DIGEST)

The DIGEST is a validated five-point ordinal scale that provides an overall rating of pharyngeal swallowing function assessed through videofluoroscopy [11]. The DIGEST includes a total score and two subscores: (i) the safety profile, derived by assigning the maximum Penetration-Aspiration scale [43] score across the different swallowing trials, (ii) the efficiency profile, derived by estimating the maximum percentage of the pharyngeal post-swallow residue. Both the total DIGEST score and the subscores range from 0 to 4 (0 = no pharyngeal dysphagia, 1 = mild, 2 = moderate, 3 = severe, 4 = life threatening).

### 4.4. Videofluoroscopic Measures

Videofluoroscopic recordings were assessed by a blinded speech and language pathologist using the Carestream software (Carestream Health, Inc.). Overall, 10 parameters were selected from the literature [9,26,44,45] for the videofluoroscopic analysis based on previous studies as salient impairments in patients with head and neck cancer [8,9,46]. They included 4 spatial measures, 2 temporal measures, and 4 ordinal variables. Definitions used to rate the 10 parameters are reported in Table 3 and Table 4. Spatial measurements were made after calibration of the digitized image to the size of a standard coin taped to the submandibular region of the patients during the swallowing study. For temporal parameters, the number of frames was counted and then transformed into seconds (number of frames: 25).

### 4.5. Statistical Analysis

Considered the small sample size, results are reported as median and interquartile (IQ) range, and non-parametric statistics were conducted. Statistical analysis was performed with the IBM SPSS Statistics 25.0^®^ package for Windows (SPSS Inc, Chicago, IL). Swallowing was judged as unsafe if the patient scored ≥2 on the DIGEST safety profile and as inefficient if the patient scored ≥2 on the DIGEST efficiency profile. The age, the time from surgery to follow-up, the T stage of the tumour, and videofluoroscopic measures were compared in: (i) patients with safe swallowing vs. patients with unsafe swallowing; (ii) patients with efficient swallowing vs. patients with inefficient swallowing. N and M stages of the tumor were constant in the included sample. The statistical significance was set at *p* < 0.05.

## 5. Conclusions

The mechanisms underlying swallowing impaired safety and efficiency have been analyzed in a group of patients who underwent an OPHL IIa+ARY. An incomplete laryngeal closure affects swallowing safety leading to laryngeal penetration and aspiration. An increased total pharyngeal transit time, a reduced UES lateral opening, and an incomplete tongue base retraction cause post-swallow pharyngeal residue, thus, reducing the swallowing efficiency. A swallowing evaluation after an OPHL type IIa+ARY should focus on the assessment of these mechanisms, in addition to the identification of signs of dysphagia. Potentially, rehabilitative and surgical approaches targeting these mechanisms may improve swallowing function in this population.

## Figures and Tables

**Table 1:** Comparisons of videofluoroscopic measures in patients with safe and unsafe swallowing.

Measure	Consistency	Safe		Unsafe		*p*
		Median	IQ Range	Median	IQ Range	
TPT (s)	solid	0.36	0.10	0.32	0.40	0.867
	semisolid	0.32	0.10	0.36	0.20	0.336
	liquid	0.42	0.11	0.36	0.04	0.536
POD (s)	solid	0.26	0.10	0.24	0.08	0.397
	semisolid	0.24	0.07	0.28	0.04	1
	liquid	0.28	0.07	0.24	0.08	0.867
POL (mm)	solid	8.5	3.5	8	3	0.867
	semisolid	12	5.5	11	5	0.152
	liquid	10	3	12	4	0.232
HMS (mm)	solid	5	10.8	2	14	0.955
	semisolid	1	3.5	6	14	0.232
	liquid	0	3.5	4	6	0.397
HMR (mm)	solid	29	7.5	26	12	0.867
	semisolid	27	14.5	32	1	0.694
	liquid	27	11.3	22	16	0.536
HVS (mm)	solid	60.5	13.5	62	4	0.867
	semisolid	60	15.5	64	1	0.613
	liquid	58	15	60	2	0.779
LC	solid	1	1.75	3	1	0.021 *
	semisolid	1	0.75	3	3	0.152
	liquid	2	2	4	1	0.021 *
EM	solid	1	1	2	0	0.121
	semisolid	1	0.75	2	1	0.152
	liquid	1	0.75	2	0	0.054
IPS	solid	0	1	0	1	0.955
	semisolid	0	0	0	0	1
	liquid	0.5	1	0	0	0.336
TBR	solid	1	0.75	2	1	0.336
	semisolid	1	0.75	2	1	0.152
	liquid	1.5	1	2	1	0.536

* *p* < 0.05; TPT: total pharyngeal transit time, POD: pharyngoesophageal segment (PES) opening duration, POL: PES opening lateral, HMS: hyoidomandibular distance during swallowing, HMR: hyoidomandibular distance at rest, HVS: hyoidovertebral distance during swallowing, LC: laryngeal closure, EM: epiglottic movement, IPS: initiation of pharyngeal swallowing, TBR: tongue base retraction.

**Table 2:** Comparisons of videofluoroscopic measures in patients with efficient and inefficient swallowing.

Measure	Consistency	Efficient		Inefficient		*p*
		Median	IQ Range	Median	IQ Range	
TPT (s)	solid	0.36	0.08	0.36	0.37	0.328
	semisolid	0.32	0.06	0.40	0.19	0.008 *
	liquid	0.40	0.14	0.36	0.06	0.776
POD (s)	solid	0.24	0.12	0.24	0.1	0.776
	semisolid	0.24	0.02	0.28	0.05	0.066
	liquid	0.28	0.10	0.28	0.07	1
POL (mm)	solid	9	4.5	8	3	0.607
	semisolid	12	4	9.5	4	0.008 *
	liquid	12	3.5	10.5	3.5	0.689
HMS (mm)	solid	6	10.5	2	12.5	0.689
	semisolid	2	5	6	12.5	0.456
	liquid	0	3	6	1	0.088
HMR (mm)	solid	28	8.5	26	14.25	0.776
	semisolid	28	11	33	13	0.328
	liquid	24	15	29	13.5	0.388
HVS (mm)	solid	64	13	60	0.75	0.328
	semisolid	66	16	63	1.05	0.607
	liquid	60	16	61	0.7	0.689
LC	solid	2	2	3	3	0.456
	semisolid	1	0.5	3.5	3	0.066
	liquid	3	3	3.5	2	0.388
EM	solid	2	1	2	1	0.776
	semisolid	1	1	2	1	0.328
	liquid	1	1	2	1	0.529
IPS	solid	0	0.5	0.5	1.25	0.328
	semisolid	0	0	0	0.25	0.864
	liquid	0	1	0	1.5	1
TBR	solid	1	0	2	0.25	0.018 *
	semisolid	1	0.5	2	0.25	0.049 *
	liquid	1	1	2	0.25	0.224

* *p* < 0.05; TPT: total pharyngeal transit time, POD: pharyngoesophageal segment opening duration, POL: pharyngoesophageal segment opening lateral, HMS: hyoidomandibular distance during swallowing, HMR: hyoidomandibular distance at rest, HVS: hyoidovertebral distance during swallowing, LC: laryngeal closure, EM: epiglottic movement, IPS: initiation of pharyngeal swallowing, TBR: tongue base retraction.

**Table 3:** Temporal and spatial videofluoroscopic measures.

Measure	Abbreviation	Unit of Measurement	Definition
Total pharyngeal transit time	TPT	s	Time from when bolus head first passes posterior nasal spine to time when bolus tale exits PES
PES opening duration	POD	s	Time from when PES first opens for bolus entry to when it first closes behind the bolus
PES opening (lateral)	POL	mm	Distance at the narrowest point of opening between C3 and C6 (upper esophageal sphincter) on lateral fluoroscopic view
Hyoidomandibular distance during swallowing	HMS	mm	Distance between the upper margin of the hyoid bone and lower margin of the mandible during swallowing
Hyoidomandibular distance at rest	HMR	mm	Distance between the upper margin of the hyoid bone and lower margin of the mandible at the standing point immediately prior to swallowing
Hyoidovertebral distance during swallowing	HVS	mm	Distance between the anterior border of vertebral spine and hyoid bone during swallowing

**Table 4:** Ordinal videofluoroscopic variables.

Ordinal Variable	Abbreviation	Operational Definitions
Laryngeal closure	LC	Ability to close the laryngeal vestibule during swallowing, assessed based on the presence or absence of air in the vestibule. Ratings: Complete and protective Complete and not protective Incomplete and protective Incomplete and not protective.
Epiglottic movement	EM	Tilting of the epiglottis during swallowing, assessed based on the contact between the epiglottis and the CAU. Ratings: Complete inversion Incomplete inversion
Initiation of pharyngeal swallowing	IPS	Site of onset of the swallowing reflex. Ratings: 0. Bolus head at posterior angle of the ramus 1. Bolus head at valleculae 2. Bolus head at posterior laryngeal surface epiglottis 3. Bolus head at pyriform sinuses 4. No appreciable initiation of swallowing at any location
Tongue base retraction	TBR	Backward movement of the tongue during swallowing, assessed based on the contact between the tongue base and the posterior pharyngeal wall. Ratings: Complete retraction Incomplete retraction
